# Gram-negative prosthetic joint infections: a retrospective multicentre European study of incidence, risk factors, and treatment outcomes

**DOI:** 10.5194/jbji-11-355-2026

**Published:** 2026-06-19

**Authors:** Ernest Famada Arboix, Arnaud Fischbacher, Matteo Carlo Maria Ferrari, Olivier Borens, Daniel Pérez-Prieto

**Affiliations:** 1 Universitat Pompeu Fabra de Barcelona, Barcelona, Spain; 2 Hospital del Mar de Barcelona, Barcelona, Spain; 3 Humanitas Clinical and Research Hospital IRCCS, Rozzano, Milan, Italy; 4 Centre Hospitalier Universitaire Vaudois, Lausanne, Switzerland

## Abstract

**Background**: Gram-negative (GN) bacteria are an increasingly recognized cause of prosthetic joint infection (PJI), accounting for 10 %–20 % of cases. However, epidemiological data from European centres remain limited. This study aimed to evaluate the incidence, risk factors, microbiological profile, and treatment outcomes of GN PJI in several European tertiary referral hospitals. **Methods**: We conducted a retrospective multicentre study including all culture-positive hip and knee PJIs diagnosed between 2014 and 2018 at three tertiary hospitals in Italy, Spain, and Switzerland. Demographic characteristics, comorbidities, surgical management, microbiological data including antimicrobial susceptibility, and treatment outcomes were analysed. Treatment success was defined as absence of persistent or recurrent infection requiring additional surgery, prosthesis removal, infection-related mortality, or long-term suppressive antibiotic therapy. **Results**: Among 780 confirmed PJIs, 71 (9.1 %) were caused by GN bacteria. The most frequent pathogens were polymicrobial infections (29.6 %), *Escherichia coli* (25.4 %), and *Pseudomonas aeruginosa* (19.7 %). GN PJI mainly affected elderly patients (median age 74 years), females (60.6 %), and those with comorbidities such as diabetes mellitus (32.4 %) and those who are overweight/obese (62 %). Hip infections were more common than knee infections (59.2 % vs. 40.8 %). Overall treatment success was 89 %. Two-stage revision showed the highest success rate of 94.8 % compared with one-stage exchange (88 %) and DAIR (81 %). Ciprofloxacin was used in 72 % of cases. **Conclusions**: GN PJI incidence was comparable to that of previous reports. These infections occur more often in elderly patients with comorbidities. Two-stage revision remains the most effective surgical strategy, and ciprofloxacin continues to be a key component of antimicrobial therapy for susceptible GN infections.

## 1 Introduction

Prosthetic joint infections (PJIs) caused by gram-negative (GN) bacteria have become an increasingly recognized and concerning cause of prosthetic failure. Bacteria resistance to antibiotics is one of the main problems that orthopaedic surgery faces nowadays. Common clinically relevant gram-negative bacteria include genera such as *Escherichia coli* and *Klebsiella* species and non-fermenters like *Pseudomonas aeruginosa* (Fantoni et al., 2019; Patel, 2023).

Gram-positive bacteria, particularly *Staphylococcus aureus*, have historically been the predominant pathogens in PJI cases as they constitute 65 % to 85 % of cases. However, the role of GN bacteria is drawing more attention as they are responsible for approximately 10 % to 20 % of PJI (Casenaz et al., 2022; Hsieh et al., 2009; Ma et al., 2023). The incidence of GN PJIs is higher in elderly patients, those with compromised immune systems, and individuals with comorbidities (Chang et al., 2023; Karczewski et al., 2024). The global trend of rising joint replacement surgeries, coupled with the ageing population in many developed countries, is likely to drive the growing incidence of these infections in the coming years. That fact is making this a public health concern of increasing importance due to the burden of the disease, especially the economic impact on governments (Sadhwani et al., 2024).

The mounting incidence of antibiotic resistance among many gram-negative pathogens like *Escherichia coli*, *Pseudomonas aeruginosa*, *Klebsiella* spp., and *Acinetobacter baumannii* only adds to the complexity. It is well recognized that these infections are becoming ever harder to manage with standard antimicrobial regimens (Gonzalez et al., 2025).

Moreover, resistance to quinolones, which are the antibiofilm antibiotic for GN PJI, has been increasing over recent years. In addition, ciprofloxacin is the only oral antibiotic available for non-fermenting gram-negative bacteria (NFGNB). When quinolone resistance occurs, it makes PJI treatment extremely difficult (Gómez-Junyent et al., 2025; Macesic et al., 2025).

Despite the growing recognition of GN pathogens in PJIs, the epidemiological impact of these infections remains poorly described, considering that joint replacement surgeries are increasing at a steady pace in Europe. Given the ageing population and the expanding number of total hip and knee replacements performed in Europe, understanding the true scale of GN PJI is essential to developing effective prevention strategies and treatment protocols. In this context, there is a need for multicentric studies that focus on the clinical characteristics, risk factors, microbial profiles, and treatment outcomes associated with GN PJI.

The primary aim of this study was to determine the incidence of gram-negative prosthetic joint infections (GN PJIs) across different European centres and to describe the treatment success rate according to the type of treatment.

Secondary objectives included the identification of major risk factors, characterization of the type of infection (early, delayed, or chronic), description of surgical management approaches, and analysis of microbiological data (including causative organisms and antibiotic resistance patterns). Additionally, we assessed the demographic and clinical characteristics of affected patients and the impact of GN PJI on patient outcomes.

## 2 Methodology

### 2.1 Study design and population

This is a retrospective study conducted in three tertiary-level referral university hospitals in three different European countries. They include the Humanitas Clinical and Research Hospital IRCCS, (Rozzano, Milan, Italy), Hospital del Mar (Barcelona, Spain), and the Centre Hospitalier Universitaire Vaudois-CHUV (Lausanne, Switzerland). All culture-positive hip and knee PJIs that were diagnosed from 2014 to 2018 in these centres were included.

### 2.2 Dataset

The dataset was constructed with data extracted from patient electronic health records from the previously mentioned centres.

To identify cases of GN PJI, multiple institutional databases were screened, including those related to hospital discharge diagnoses, surgical site infections, and microbiological results.

Medical records of all potentially eligible patients were subsequently reviewed to confirm the diagnosis of GN PJI according to the established criteria of Zimmerli et al. (2004). During this process, relevant demographic data, clinical characteristics, and risk factors were systematically collected using a standardized data extraction approach.

Information related to joint infections, prosthetic joint infections, prosthetic debridement, and partial or total revisions was extracted from those data.

Only patients with microbiologically confirmed GN PJI, defined by positive cultures for gram-negative organisms, were included in the final analysis.

Exclusion criteria were patients under 18 years of age, patients that did not have a confirmed diagnosis of a PJI, and patients that were unable to fully understand the nature and purpose of the study.

The final sample consisted of 780 patients with a confirmed PJI.

### 2.3 Statistical analysis

The descriptive analysis was performed based on each type of variable. Qualitative variables were encoded numerically and were expressed as a percentage (%) of the total sample. Continuous quantitative variables were expressed as means (range), while ordinal quantitative variables were reported as mean values. A comparative analysis was conducted using the $\chi^{2}$ (chi-square) test.

All statistical analyses were conducted with the IBM SPSS Statistics 31.0.0 software package.

## 3 Results

**Figure 1:**
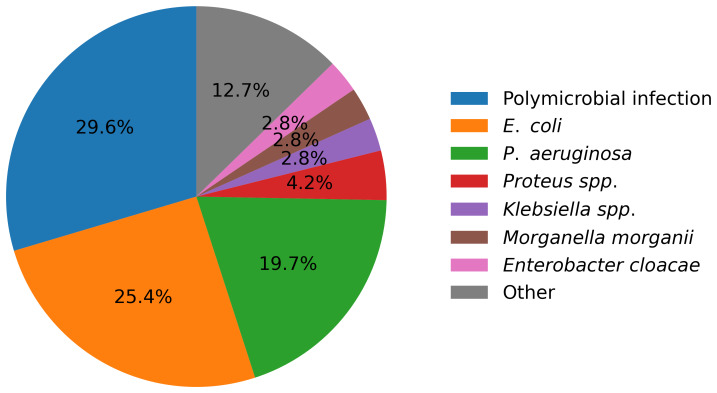
Microorganism infection distribution.

In the present cohort, 71 (9.1 %) out of the 780 PJIs were caused by GN bacteria (Table 1). Polymicrobial infection was the most prevalent with 29.6 %, followed by *Escherichia coli* at 25.4 % and *Pseudomonas aeruginosa* at 19.7 % (Table 2, Fig. 1). GN PJIs were more common in females (60.6 %) than males (39.4 %). Comorbidities were also remarkable in the present sample: diabetes mellitus was diagnosed in 32.4 % of the patients, and 62 % were overweight. The median age in the sample was 74 years with a median ASA score of 3.

**Table 1:** Population demography.

Total PJIs	N=780
GN PJI	71 (9.1 %)
Age	
Mean (SD)	73.31 (11.13)
Median ( $Q_{1}$ , $Q_{3}$ )	74.0 (65.0, 82.0)
Min, max	42.0, 93.4
N (% non-missing)	71 (100.0 %)
Gender	
Male	28 (39.4 %)
Female	43 (60.6 %)
Arthroplasty	
Hip	42 (59.2 %)
Knee	29 (40.8 %)
Overweight	
Y	44 (62.0 %)
ASA	
2	32 (45.1 %)
3	36 (50.7 %)
4	3 (4.2 %)
Diabetes	23 (32.4 %)
Immunosuppression	6 (8.5 %)
COPD	15 (21.1 %)
Infection type	
Acute	14 (19.7 %)
Acute hematogenous	14 (19.7 %)
Chronic	43 (60.6 %)
Resistances	20 (28.2 %)
Resistant ESBL^1^	11 (15.5 %)
Resistant ciprofloxacin	12 (16.9 %)
Treatment	
One stage	8 (11.3 %)
Two stages	39 (54.9 %)
DAIR^2^	21 (29.6 %)
Implant removal without replacement	3 (4.2 %)
Outcome	
Relapse	8 (11.3 %)
Cured	63 (88.7 %)

^1^ ESBL (extended-spectrum betalactamases). ^2^ DAIR (debridation antibiotic and implant retention).

**Table 2:** Microorganism distribution.

Polimicrobial infection	21 (29.6 %)
*E. coli*	18 (25.4 %)
*Pseudomonas aeruginosa*	14 (19.7 %)
*Proteus vulgaris*	1 (1.4 %)
*Proteus mirabillis*	2 (2.8 %)
*Klebsiella pneumonie*	1 (1.4 %)
*Morganella morganii*	2 (2.8 %)
*Enter obacter cloacae*	2 (2.8 %)
*Fusobacterium nucleatum*	1 (1.4 %)
*Klebsiella oxytoca*	1 (1.4 %)
*Cuprividus pauculus*	2 (2.8 %)
*Neisseria flavescens*	1 (1.4 %)
*Pantoea agglomerans*	1 (1.4 %)
*Ralstonia pickettii*	2 (2.8 %)
*Ralstonia insidiosa*	1 (1.4 %)
*Campylocabter fetus*	1 (1.4 %)

Within the GN group consisting of 71 cases, 59.2 % had a hip infection, and 40.8 % had a knee infection.

Two-stage revision was performed in 54.9 % of the patients, debridement antibiotic and implant retention (DAIR) in 29.6 %, one-step exchange in 11.3 %, and implant removal without replacement in 4.2 %.

The overall treatment success rate was 89 %. Interestingly, the success rate was better for the two-step replacement (94.8 %) when compared to the one-step replacement (88 %) and (DAIR) with a success rate of 81 % ( p=0.187 ) (Table 2).

The median duration of antibiotic treatment in the success group was 72 d, and the duration was 80 d in the failure group. Ciprofloxacin was used as oral treatment in 51 cases (72 %). No ciprofloxacin resistances were observed in the relapsed group, whereas there were 12 resistant cases in the cured group (19 %). Ciprofloxacin was used in seven out of eight relapsed cases.

Infections caused by *Escherichia coli* were associated with a lower success rate (83 %) than that of *Pseudomonas aeruginosa* (93 %) and polymicrobial infections (90 %) ( p=0.358 ).

Finally, the success rate was better in knee PJI compared to hip PJI (97 % vs. 83 %, p=0.121 ) and in females compared to males (93 % vs. 82 %, p=0.121 ) (Table 3).

## 4 Discussion

The most interesting finding of the present study is that it confirms the reported trend of PJI caused by GN bacteria. In the present cohort they accounted for 9.1 % of all PJI cases, consistent with large series reporting a 11 %–15 % prevalence rate for GN PJI (Hsieh et al., 2009). The most common causative organisms were *Escherichia coli* and *Pseudomonas aeruginosa*, with a notable number of cases being polymicrobial, which is consistent with the higher rate of polymicrobial gram-negative PJI described in the literature (Fantoni et al., 2019).

GN PJIs were more frequent in females and in older patients. They also had high rates of comorbidities such as diabetes and high body mass index. These findings are consistent with prior studies showing being elderly and having comorbidities as risk factors for gram-negative PJI. Karczewski et al. (2024) highlighted that the mean age of patients with GN PJIs was 77 years, and a significant proportion had comorbidities such as diabetes mellitus (Karczewski et al., 2024). Additionally, Chang et al. (2023) noted that gram-negative bacteria were more frequently isolated in patients with chronic PJIs that often have underlying health issues (Chang et al., 2023).

**Table 3:** Population demography description by outcome.

	Relapse	Cured	Total	p value
	( N=8 )	( N=63 )	( N=71 )	
Age				0.094
Mean (SD)	79.97 (7.55)	72.46 (11.26)	73.31 (11.13)	
Median ( $Q_{1}$ , $Q_{3}$ )	78.8 (76.0, 83.8)	72.0 (64.0, 82.0)	74.0 (65.0, 82.0)	
Min, max	69.0, 93.4	42.0, 92.0	42.0, 93.4	
N (% non-missing)	8 (100.0 %)	63 (100.0 %)	71 (100.0 %)	
ATB duration (days)				0.056
Mean (SD)	85.43 (20.65)	69.83 (47.97)	71.39 (46.12)	
Median ( $Q_{1}$ , $Q_{3}$ )	90.0 (60.0, 90.0)	66.0 (42.0, 90.0)	68.0 (48.0, 90.0)	
Min, max	60.0, 120.0	28.0, 400.0	28.0, 400.0	
N (% non-missing)	7 (87.5 %)	63 (100.0 %)	70 (98.6 %)	
Sex				0.249
Male	5 (62.5 %)	23 (36.5 %)	28 (39.4 %)	
Female	3 (37.5 %)	40 (63.5 %)	43 (60.6 %)	
Arthroplasty				0.130
Hip	7 (87.5 %)	35 (55.6 %)	42 (59.2 %)	
Knee	1 (12.5 %)	28 (44.4 %)	29 (40.8 %)	
Resistance	6 (75.0 %)	45 (71.4 %)	51 (71.8 %)	0.601
Resistance ESBL	2 (25.0 %)	9 (14.3 %)	11 (15.5 %)	0.336
Resistance ciprofloxacin	0 (0.0 %)	12 (19.0 %)	12 (16.9 %)	0.657
Infection type				1.000
Acute	2 (25.0 %)	12 (19.0 %)	14 (19.7 %)	
Acute hematogenous	2 (25.0 %)	12 (19.0 %)	14 (19.7 %)	
Chronic	4 (50.0 %)	39 (61.9 %)	43 (60.6 %)	
*Pseudomonas aeruginosa*	1 (12.5 %)	13 (20.6 %)	14 (19.7 %)	1.000
*Enterobacter cloacae*	0 (0.0 %)	2 (3.2 %)	2 (2.8 %)	0.409
*E. coli*	3 (37.5 %)	15 (23.8 %)	18 (25.4 %)	1.000
*Campylocabter fetus*	0 (0.0 %)	1 (1.6 %)	1 (1.4 %)	1.000
*Polimicrobial*	2 (25.0 %)	19 (30.2 %)	21 (29.6 %)	1.000
*Klebsiella pneumonie*	0 (0.0 %)	1 (1.6 %)	1 (1.4 %)	0.214
*Morganella morganii*	1 (12.5 %)	1 (1.6 %)	2 (2.8 %)	1.000
*Proteus vulgaris*	0 (0.0 %)	1 (1.6 %)	1 (1.4 %)	1.000
*Proteus mirabillis*	0 (0.0 %)	2 (3.2 %)	2 (2.8 %)	1.000
*Fusobacterium nucleatum*	0 (0.0 %)	1 (1.6 %)	1 (1.4 %)	0.113
*Klebsiella oxytoca*	1 (12.5 %)	0 (0.0 %)	1 (1.4 %)	1.000
*Cuprividus pauculus*	0 (0.0 %)	2 (3.2 %)	2 (2.8 %)	1.000
*Neisseria flavescens*	0 (0.0 %)	1 (1.6 %)	1 (1.4 %)	1.000
*Pantoea agglomerans*	0 (0.0 %)	1 (1.6 %)	1 (1.4 %)	1.000
*Ralstonia pickettii*	0 (0.0 %)	2 (3.2 %)	2 (2.8 %)	1.000
*Ralstonia insidiosa*	0 (0.0 %)	1 (1.6 %)	1 (1.4 %)	0.128
Treatment				0.047
One stage	1 (12.5 %)	7 (11.1 %)	8 (11.3 %)	
Two stages	2 (25.0 %)	37 (58.7 %)	39 (54.9 %)	
DAIR^*^	4 (50.0 %)	17 (27.0 %)	21 (29.6 %)	

**Table 3:** Continued.

	Relapse	Cured	Total	p value
	( N=8 )	( N=63 )	( N=71 )	
Implant removal without replacement	1 (12.5 %)	2 (3.2 %)	3 (4.2 %)	
Overweight	2 (25.0 %)	42 (66.7 %)	44 (62.0 %)	0.399
ASA				0.708
2	3 (37.5 %)	29 (46.0 %)	32 (45.1 %)	
3	4 (50.0 %)	32 (50.8 %)	36 (50.7 %)	
4	1 (12.5 %)	2 (3.2 %)	3 (4.2 %)	
Diabetes	3 (37.5 %)	20 (31.7 %)	23 (32.4 %)	1.000
Immunosuppression	0 (0.0 %)	6 (9.5 %)	6 (8.5 %)	0.189
COPD	0 (0.0 %)	15 (23.8 %)	15 (21.1 %)	0.105
Positive blood cultures	3 (37.5 %)	8 (12.9 %)	11 (15.7 %)	0.19

^*^ DAIR (debridation antibiotic and implant retention).

The predominance of hip PJI over knee PJI is also in line with published data and in line with clinical thought due to its proximity to the intestinal and urogenital tracts (Rodríguez-Pardo et al., 2014).

Regarding management strategies, the most common surgical approach was a two-stage replacement. It was followed by DAIR and the one-stage replacement. Resection arthroplasty was of more marginal utility. The overall treatment success rate was high (89 %), with two-stage replacement yielding the highest success rate at 95 % compared with DAIR standing at 81 % and the one-stage replacement at 87 %. This is consistent with the literature, which shows superior outcomes for two-stage exchange in GN PJI when compared with DAIR and the one-stage replacement (Gómez-Junyent et al., 2025).

The Infectious Diseases Society of America (IDSA) and the American Society for Microbiology (ASM) recommend a two-stage exchange as the standard for chronic GN PJI, with DAIR reserved for acute cases with stable implants (Anon, 2019). However, for the European standards, one stage is a valid option for selected cases. Even though the two-stage approach revealed better infection control outcomes in the present cohort, patient characteristics have not been assessed as confounders (Sousa et al., 2023).

Ciprofloxacin was used in 44 patients in the success group and omitted in one of eight relapsed cases in this study. That success supports the findings in previous literature that fluoroquinolones are associated with higher success rates in multi-drug-resistent gram-negative bacteria (MDR GNB) prosthetic joint infections due to their superior anti-biofilm activity and clinical outcomes with non-resistant bacteria (Gómez-Junyent et al., 2025; Hanssen et al., 2024). This aligns with the established consensus that fluoroquinolones, particularly ciprofloxacin, are the preferred oral agents for susceptible MDR GNB PJI. Their pharmacokinetic properties and biofilm penetration capacity contribute significantly to improved outcomes in device-related infections (Gómez-Junyent et al., 2025; Hanssen et al., 2024).

The absence of ciprofloxacin in some relapsed cases likely reflects resistance or intolerance, leaving limited oral options available when fluoroquinolone susceptibility is lost. In such scenarios, alternative agents like delafloxacin may be considered as it maintains activity against some ciprofloxacin-resistant strains. However, clinical data for delafloxacin in PJI are limited, and its role has not yet been established (Bassetti et al., 2018; Gómez-Junyent et al., 2025).

The data reinforce the importance of individualized therapy for resistant strains that is based on susceptibility testing and use of newer quinolones.

The prevalence of fluoroquinolone resistance in Europe in the context of prosthetic infections varies depending on the microorganism involved and the region in which we treat these infections. The most recent data show that resistance exceeds 20 % in key pathogens such as *Escherichia coli*, *Klebsiella pneumoniae*, and *Acinetobacter* spp., especially in strains associated with nosocomial and multi-drug-resistant infections (Gómez-Junyent et al., 2025; Hanssen et al., 2024).

Success rates were higher in females and in knee PJI, but these differences were not significant. This may reflect differences in host factors or surgical complexity, but no definitive conclusions can be drawn from our results (Martínez-Pastor et al., 2009; Tai et al., 2022).

In summary, this multicentre European study provides a descriptive overview of gram-negative prosthetic joint infections and is broadly consistent with previously published evidence. This study also adds a valuable multicentre perspective across different European settings, an area that remains insufficiently explored.

This study also has some limitations. It is a retrospective, descriptive study with an inherent selection bias. Although we standardized the diagnostic criteria following the Zimmerli et al. (2004) criteria, it is not possible to control all the potentially inherent biases in a retrospective clinical cohort study. Furthermore, the demographic and clinical characteristics of the cohort (age, comorbidity burden, and chronicity of infection) may not reflect the characteristics of other regions not studied, which limits the study's external validity. The primarily descriptive design and the absence of multivariable analysis limit the strength of causal inferences; therefore, the findings should be interpreted as exploratory. Nevertheless, the observed variability in microbiological patterns and antibiotic susceptibility across centres may have potential implications for clinical decision-making.

## 5 Conclusions

Gram-negative PJIs had a similar incidence to those in studies prior to this one. They are more frequent in the elderly; females; and patients with comorbidities like diabetes, patients who are overweight, and/or patients who have immunosuppressive pathologies. The two-stage exchange remains the most effective surgical strategy. Pathogens like *E. coli* seem to have lower cure rates. Ciprofloxacin remains a cornerstone of antimicrobial therapy for susceptible GN PJI. These findings support individualized treatment approaches based on patient characteristics, pathogen susceptibility, and infection chronicity.

## Data Availability

No data sets were used in this article.
